# Guidelines for Genome-Scale Analysis of Biological Rhythms

**DOI:** 10.1177/0748730417728663

**Published:** 2017-11-03

**Authors:** Michael E. Hughes, Katherine C. Abruzzi, Ravi Allada, Ron Anafi, Alaaddin Bulak Arpat, Gad Asher, Pierre Baldi, Charissa de Bekker, Deborah Bell-Pedersen, Justin Blau, Steve Brown, M. Fernanda Ceriani, Zheng Chen, Joanna C. Chiu, Juergen Cox, Alexander M. Crowell, Jason P. DeBruyne, Derk-Jan Dijk, Luciano DiTacchio, Francis J. Doyle, Giles E. Duffield, Jay C. Dunlap, Kristin Eckel-Mahan, Karyn A. Esser, Garret A. FitzGerald, Daniel B. Forger, Lauren J. Francey, Ying-Hui Fu, Frédéric Gachon, David Gatfield, Paul de Goede, Susan S. Golden, Carla Green, John Harer, Stacey Harmer, Jeff Haspel, Michael H. Hastings, Hanspeter Herzel, Erik D. Herzog, Christy Hoffmann, Christian Hong, Jacob J. Hughey, Jennifer M. Hurley, Horacio O. de la Iglesia, Carl Johnson, Steve A. Kay, Nobuya Koike, Karl Kornacker, Achim Kramer, Katja Lamia, Tanya Leise, Scott A. Lewis, Jiajia Li, Xiaodong Li, Andrew C. Liu, Jennifer J. Loros, Tami A. Martino, Jerome S. Menet, Martha Merrow, Andrew J. Millar, Todd Mockler, Felix Naef, Emi Nagoshi, Michael N. Nitabach, Maria Olmedo, Dmitri A. Nusinow, Louis J. Ptáček, David Rand, Akhilesh B. Reddy, Maria S. Robles, Till Roenneberg, Michael Rosbash, Marc D. Ruben, Samuel S.C. Rund, Aziz Sancar, Paolo Sassone-Corsi, Amita Sehgal, Scott Sherrill-Mix, Debra J. Skene, Kai-Florian Storch, Joseph S. Takahashi, Hiroki R. Ueda, Han Wang, Charles Weitz, Pål O. Westermark, Herman Wijnen, Ying Xu, Gang Wu, Seung-Hee Yoo, Michael Young, Eric Erquan Zhang, Tomasz Zielinski, John B. Hogenesch

**Affiliations:** 1 Division of Pulmonary and Critical Care Medicine, Washington University School of Medicine, St. Louis, Missouri, USA; 2 Department of Biology and Howard Hughes Medical Institute, Brandeis University, Waltham, Massachusetts, USA; 3 Department of Neurobiology, Northwestern University, Evanston, Illinois, USA; 4 Division of Sleep Medicine, Department of Medicine, University of Pennsylvania Perelman School of Medicine, Philadelphia, USA; 5 Center for Integrative Genomics, Génopode, University of Lausanne, Lausanne, Switzerland; 6 Vital-IT, Swiss Institute of Bioinformatics, Lausanne, Switzerland; 7 Department of Biomolecular Sciences, Weizmann Institute of Science, Rehovot, Israel; 8 Institute for Genomics and Bioinformatics, University of California, Irvine, USA; 9 Department of Biology, University of Central Florida, Orlando, USA; 10 Department of Biology, Texas A&M University, College Station, USA; 11 Department of Biology, New York University, New York, USA; 12 Institute of Pharmacology and Toxicology, University of Zürich, Switzerland; 13 Laboratorio de Genética del Comportamiento, Fundación Instituto Leloir, IIBBA-CONICET, Buenos Aires, Argentina; 14 Department of Biochemistry and Molecular Biology, University of Texas Health Science Center, Houston, USA; 15 Department of Entomology and Nematology, University of California, Davis, USA; 16 Computational Systems Biochemistry, Max-Planck Institute of Biochemistry, Martinsried, Germany; 17 Department of Molecular and Systems Biology, Geisel School of Medicine at Dartmouth, Hanover, New Hampshire, USA; 18 Department of Pharmacology and Toxicology, Morehouse School of Medicine, Atlanta, Georgia, USA; 19 Surrey Sleep Research Centre, University of Surrey, Guildford, UK; 20 The University of Kansas Medical Center, University of Kansas, Kansas City, USA; 21 John A. Paulson School of Engineering and Applied Sciences, Harvard University, Boston, Massachusetts, USA; 22 Department of Biological Sciences and Eck Institute for Global Health, University of Notre Dame, Notre Dame, Indiana, USA; 23 Institute of Molecular Medicine, McGovern Medical School, UT Health Houston, Houston, Texas, USA; 24 Department of Physiology and Functional Genomics, University of Florida College of Medicine, Gainesville, USA; 25 Systems Pharmacology and Translational Therapeutics, University of Pennsylvania Perelman School of Medicine, Philadelphia, USA; 26 Department of Mathematics, University of Michigan, Ann Arbor, USA; 27 Department of Pediatrics, Cincinnati Children’s Hospital Medical Center, Cincinnati, Ohio, USA; 28 Kavli Institute for Fundamental Neuroscience, Weill Institute of Neuroscience, Department of Neurology, University of California, San Francisco, USA; 29 Department of Diabetes and Circadian Rhythms, Nestlé Institute of Health Sciences, Lausanne, Switzerland; 30 Department of Endocrinology & Metabolism, Academic Medical Center, Amsterdam, the Netherlands; 31 Center for Circadian Biology and Division of Biological Sciences, University of California, San Diego, La Jolla, USA; 32 Department of Neuroscience, University of Texas Southwestern Medical Center, Dallas, USA; 33 Department of Mathematics, Duke University, Durham, North Carolina, USA; 34 Department of Plant Biology, University of California, Davis, USA; 35 Medical Research Council Laboratory of Molecular Biology, Cambridge, UK; 36 Institute for Theoretical Biology, Charité-Universitätsmedizin Berlin, Germany; 37 Department of Biology, Washington University in St. Louis, Missouri, USA; 38 Department of Biomedical Informatics, Vanderbilt University School of Medicine, Nashville, Tennessee, USA; 39 Department of Biological Sciences, Rensselaer Polytechnic Institute, Troy, New York, USA; 40 Department of Biology, University of Washington, Seattle, USA; 41 Department of Biological Sciences, Vanderbilt University, Nashville, Tennessee, USA; 42 Department of Cell and Molecular Biology, The Scripps Research Institute, University of California, San Diego, La Jolla, USA; 43 Department of Physiology and Systems Bioscience, Kyoto Prefectural University of Medicine, Japan; 44 Division of Sensory Biophysics, The Ohio State University, Columbus, USA; 45 Laboratory of Chronobiology, Charité Universitätsmedizin Berlin, Germany; 46 Department of Molecular Medicine, The Scripps Research Institute, La Jolla, California, USA; 47 Department of Mathematics and Statistics, Amherst College, Amherst, Massachusetts, USA; 48 Department of Biology, University of Missouri–St. Louis, USA; 49 Department of Cell Biology, College of Life Sciences at Wuhan University, China; 50 Department of Biological Sciences, University of Memphis, Tennessee, USA; 51 Department of Biochemistry and Cell Biology, Geisel School of Medicine at Dartmouth, Hanover, New Hampshire, USA; 52 Centre for Cardiovascular Investigations, Department of Biomedical Sciences, University of Guelph, Guelph, Ontario, Canada; 53 Institute of Medical Psychology, Faculty of Medicine, LMU Munich, Germany; 54 SynthSys and School of Biological Sciences, University of Edinburgh, UK; 55 Donald Danforth Plant Science Center, St. Louis, Missouri, USA; 56 The Institute of Bioengineering, School of Life Sciences, Ecole Polytechnique Fédérale de Lausanne, Switzerland; 57 Department of Genetics and Evolution, University of Geneva, Switzerland; 58 Department of Cellular and Molecular Physiology, Department of Genetics, Kavli Institute for Neuroscience, Yale School of Medicine, New Haven, Connecticut, USA; 59 Department of Genetics, University of Seville, Spain; 60 Department of Neurology, University of California, San Francisco, USA; 61 Warwick Systems Biology and Mathematics Institute, University of Warwick, Conventry, UK; 62 The Francis Crick Institute, London, UK, and UCL Institute of Neurology, Queen Square, London, UK; 63 Centre for Immunity, Infection and Evolution, University of Edinburgh, UK; 64 Department of Biochemistry and Biophysics, University of North Carolina, Chapel Hill, USA; 65 Department of Biological Chemistry, Center for Epigenetics and Metabolism, University of California, Irvine, USA; 66 Howard Hughes Medical Institute, University of Pennsylvania Perelman School of Medicine, Philadelphia, USA; 67 Department of Microbiology, University of Pennsylvania Perelman School of Medicine, Philadelphia, USA; 68 Chronobiology, Faculty of Health and Medical Sciences, University of Surrey, Guildford, UK; 69 Department of Psychiatry, Douglas Mental Health University Institute, McGill University, Montreal, Canada; 70 Howard Hughes Medical Institute, Department of Neuroscience, University of Texas Southwestern Medical Center, Dallas, USA; 71 Department of Systems Pharmacology, Graduate School of Medicine, The University of Tokyo, Tokyo, Japan Laboratory for Synthetic Biology, RIKEN Quantitative Biology Center, Osaka, Japan; 72 Center for Circadian Clocks, Soochow University, Suzhou, Jiangsu, China; 73 Department of Neurobiology, Harvard Medical School, Boston, Massachusetts, USA; 74 Institute of Genetics and Biometry, Leibniz Institute for Farm Animal Biology, Dummerstorf, Germany; 75 Biological Sciences and Institute for Life Sciences, University of Southampton, UK; 76 Cam-Su GRC, Soochow University, Suzhou, China; 77 Laboratory of Genetics, Rockefeller University, New York, New York, USA; 78 National Institute of Biological Sciences, Beijing, China

**Keywords:** circadian rhythms, diurnal rhythms, computational biology, functional genomics, systems biology, guidelines, biostatistics, RNA-seq, ChIP-seq, proteomics, metabolomics

## Abstract

Genome biology approaches have made enormous contributions to our understanding of biological rhythms, particularly in identifying outputs of the clock, including RNAs, proteins, and metabolites, whose abundance oscillates throughout the day. These methods hold significant promise for future discovery, particularly when combined with computational modeling. However, genome-scale experiments are costly and laborious, yielding “big data” that are conceptually and statistically difficult to analyze. There is no obvious consensus regarding design or analysis. Here we discuss the relevant technical considerations to generate reproducible, statistically sound, and broadly useful genome-scale data. Rather than suggest a set of rigid rules, we aim to codify principles by which investigators, reviewers, and readers of the primary literature can evaluate the suitability of different experimental designs for measuring different aspects of biological rhythms. We introduce CircaInSilico, a web-based application for generating synthetic genome biology data to benchmark statistical methods for studying biological rhythms. Finally, we discuss several unmet analytical needs, including applications to clinical medicine, and suggest productive avenues to address them.

It has become a cliché to comment on the rapid growth of “–omics” technologies in biomedical sciences over the past 20 years. Nevertheless, it is difficult to overstate the transformative impact that genome-scale technologies are having on the practice of modern biology, notably including transcriptional, proteomic, and metabolomic profiling (Fig. 1A). These analytical approaches have had a substantial impact on the study of circadian rhythms (Fig. 1B), particularly since biological rhythms are ubiquitous at every level of organismal physiology and are seemingly custom made for large-scale analysis. Systems biology approaches offer enormous opportunities to gain insight into the nature of biological rhythms, but they also create unique challenges in properly collecting and interpreting large data sets.

**Figure 1.**
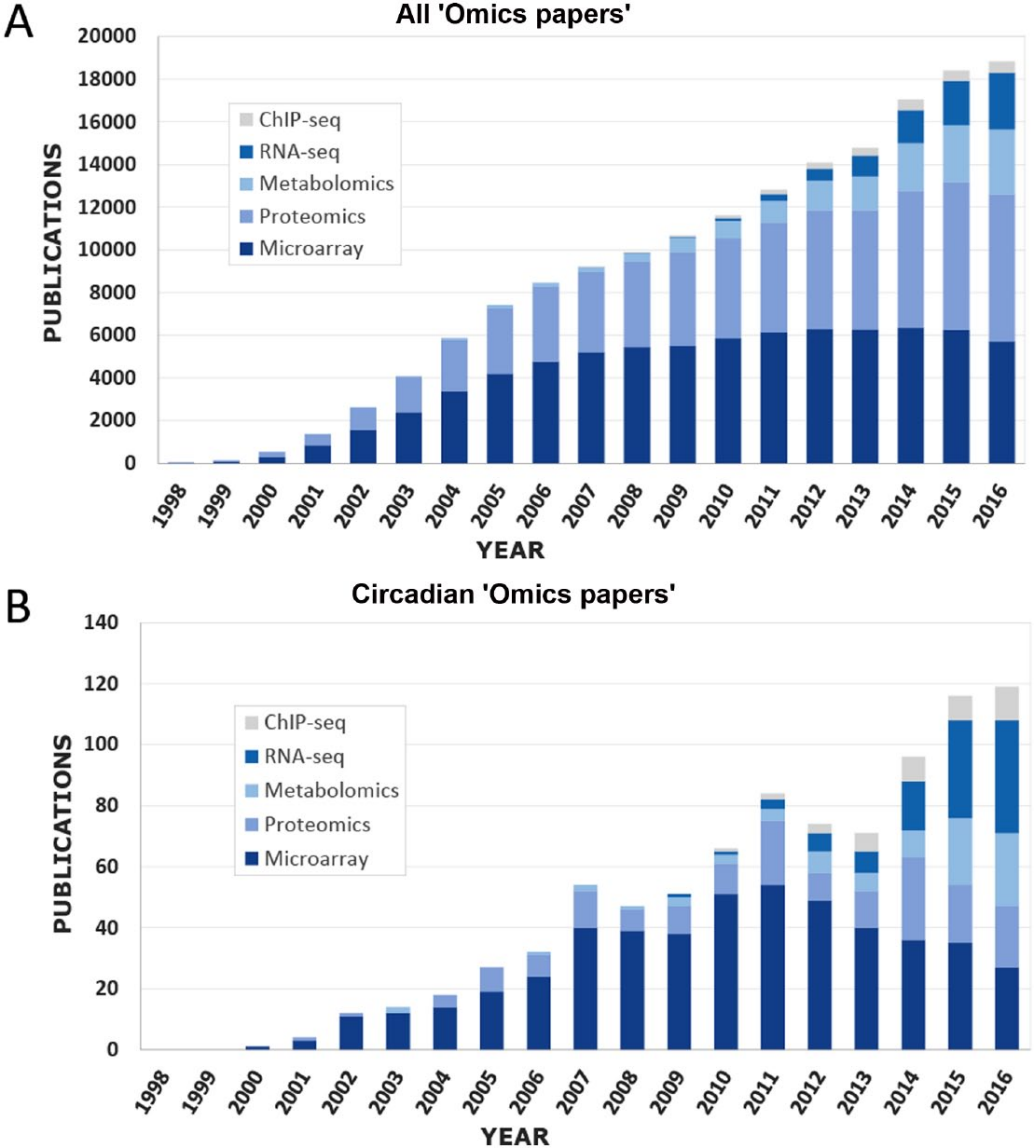
The use of systems biology approaches has increased dramatically in the past 20 years. (A) Annual number of publications available on PubMed that contain the keywords “ChIP-seq,” “RNA-seq,” “Metabolomics,” “Proteomics,” and/or “Microarray.” These numbers were obtained directly from PubMed’s “Results by Year” section. (B) A Boolean search was used to filter the number of publications containing the chosen keyword combined with the term “circadian,” “clock,” or both. Both plots depict an increase in the use of functional genomics approaches in biology over the past 5 years, in particular the use of RNA-seq, ChIP-Seq, and metabolomics.

Here, we set out to codify unifying principles for genome-scale analyses of biological rhythms. We confine our discussion to the analysis of rhythmic abundance of RNAs, proteins, and metabolites, as well as rhythmic occupancy of DNA by proteins. These guidelines also apply to the study of related processes such as promoter activity (Liu et al., 1995). We do not discuss the analysis of other large data sets, including genomewide association studies, mutagenesis and cell-based screens, or the use of “wearables” that track physiological parameters. All 3 unquestionably produce large data sets and are important for the field, but they present technical challenges beyond our scope here. We further restrict ourselves to discussing general principles. When appropriate, we refer the reader to more detailed discussions of critical topics such as sample collection and statistical benchmarking. We emphasize that these guidelines are current at the time they were written but should not be used as hard rules to replace informed peer review. Instead, we hope that this article will formalize a consensus regarding best practices for generation and analysis of large-scale biological rhythms data sets and thereby increase the rigor and reproducibility of research in our field.

## Recommendations

### Experimental Design

Before collecting large-scale data on rhythmic processes, careful consideration should be given to which questions the data are intended to answer. For example, an experiment aimed at discovery (i.e., a list of cycling transcripts/proteins/metabolites that will be validated with other methods) can be done with a less stringent design than experiments aimed at comprehensive identification of all cycling entities, along with accurate estimation of their waveform, phase, and amplitude (e.g., Zhang et al., 2014). Key considerations include the precision and accuracy of the measurements being made, the degree of rhythmicity in the data set, and the signal-to-noise ratio of the rhythms. These factors also depend on the specific model system under study and the measurement technology. Even closely related experimental approaches (e.g., RNA sequencing [RNA-seq] and chromatin immunoprecipitation sequencing [ChIP-seq]), influence the experimental design in important ways. We begin our discussion of experimental design with specific recommendations for discovery-based approaches, since it is the most common application of systems biology techniques to biological rhythms and illustrates the key principles of experimental design. We conclude this section by discussing variations on this theme.

By definition, biological rhythms repeat. We therefore recommend collecting at least 2 complete cycles of data when detecting rhythmicity (i.e., 48 h for collections under constant conditions). The guiding principle behind this recommendation is that when identifying a rhythmic process, one would like to observe both the peak and trough repeat at least once. Simulations show that collecting fewer than 2 cycles in a time series makes the resulting data sensitive to outliers and can dramatically increase the number of false-negatives (see the “Synthetic Data for Benchmarking” section). A key caveat is that it is often difficult in human and some model organisms to collect across more than 1 circadian cycle. In such cases, increasing the number of replicates may offset the disadvantage of a shorter time series.

When looking for processes regulated solely by the circadian clock, it is best to isolate your experimental organism from external zeitgebers. In many cases, this means constant darkness (DD) and constant temperature, although for photosynthetic organisms, constant light (LL) is the conventional manipulation for studying intrinsic rhythmicity. For human studies, consistent conditions (e.g., regular meal, exercise, and bed times) are essential. For some tissues, other external stimuli (e.g., food) are at least as important zeitgebers as light. Many rhythms are damped after external stimuli are removed. Therefore, we recommend sampling consecutive days after releasing entrained organisms into constant conditions. Studies of synchronized in vitro cultures should begin their sample collections 24 h after cessation of the synchronizing stimulus to minimize the impact of immediate early gene expression. This transient burst and then decay in expression of select genes in the first 24 h can erroneously look like part of the circadian cycle. In constant DD or LL conditions, circadian period length can differ from 24 h. For example, after 3 days in DD, a short period organism (~23.5 h) will start locomotor activity and other behaviors 1.5 h earlier than wild-type controls. As such, experiments in constant conditions should tune all statistical tests to the organism’s empirically determined period length.

If experiments are done under driven (e.g., light:dark [LD]) conditions, performing experiments over consecutive days is the same as collecting additional replicates on the first day, as clocks reset each day to light. Therefore, when searching for rhythms under driven (LD) conditions, 2 or more independent days of sample collection can be treated as biological replicates. This experimental design can be advantageous when the focus of the study is rhythmicity under natural conditions, rather than isolated outputs of the circadian clock. Nonconsecutive days may be used as replicates in LD; in fact, it can be beneficial to separate the collection of replicate samples in LD by as much as a week to reduce batch effects.

**Figure 2.**
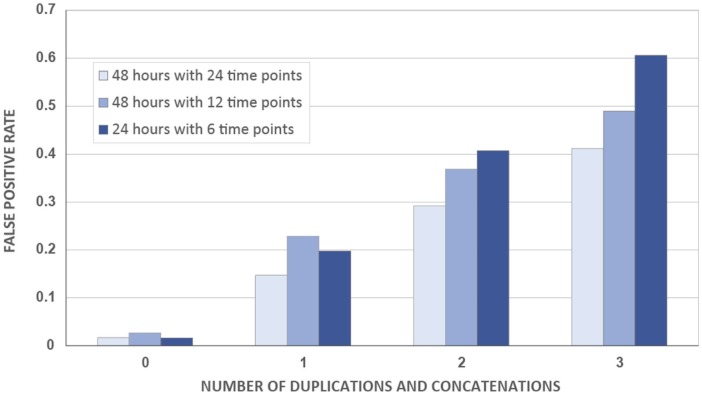
Duplicating and concatenating time-series data results in unacceptable false-positive rates. Duplicating and concatenating data to generate an artificially long time series eliminates statistical independence of samples. To empirically investigate the consequences of this manipulation, a randomly generated test set containing 1000 arrhythmic time series composed entirely of Gaussian noise was used to compare the effects of duplication and concatenation on the false-positive rate. The first simulated experiment had a duration of 48 h, with a sampling interval of 2 h. The second simulation was composed of every other time point from the first run, which resulted in a data set with a duration of 48 h and a sampling interval of 4 h. The third simulation was generated using the first half of the second run, which produced a data set with a duration of 24 h and a sampling interval of 4 h. JTK_Cycle was used to assess rhythmicity with a statistical threshold of adjusted *p* < 0.05 considered a “hit”. Without concatenation, each run produced conservative false-positive rates, with the number of hits less than 2% in every scenario. Adding the first concatenation increased the false-positive rate by a minimum of 8-fold. The second concatenation altered the initial false-positive rate by a minimum of 13-fold, and the third concatenation increased the false-positive rate by 18-fold compared with the initial rate.

Data should never be duplicated and concatenated prior to statistical testing (Text Box 1.1). By this, we mean the deliberate copying and pasting of data to artificially generate longer time series. Statistical analysis assumes the independence of each data point. Duplication of data points renders them no longer independent, and statistical tests are necessarily compromised. Furthermore, simulations show that duplicated/concatenated data have dramatically elevated false-positive rates (Fig. 2). A more subtle violation of data independence is seen when technical replicates (e.g., repeated microarrays on the same sample) are treated as biological replicates (i.e., completely independent biological specimens). In this case, natural biological variation will artificially repeat across the technical replicates, and *p* values will be inappropriately more significant. Further, we caution investigators against double plotting genome-scale time-series data, even when presented in figures for visual purposes. Although double plotting can increase clarity, it risks misleading the reader about the experimental design.

**Figure:**
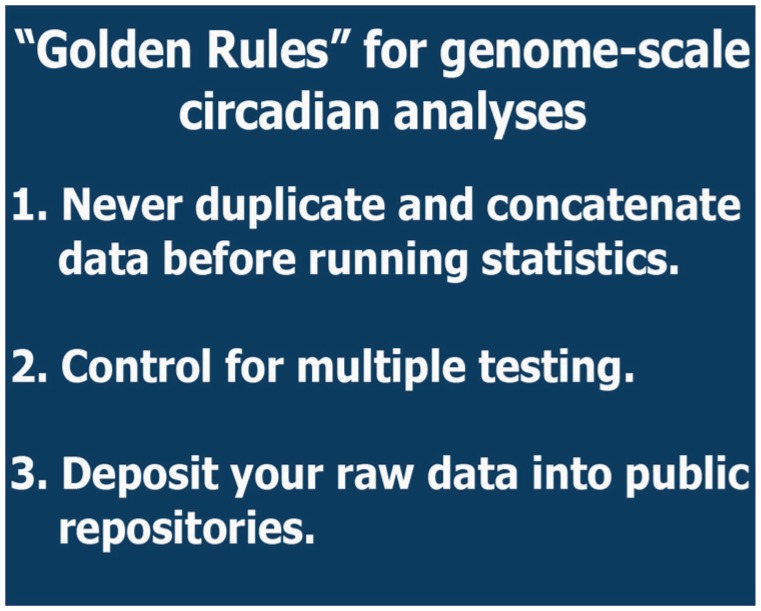


Historically, the majority of circadian data were collected with 4-h sampling resolution. This experimental design dates back to the 1980s, when Northern and Western blot assays were common. These experiments typically focused on a few relatively high-amplitude core clock or output genes/proteins. When few entities are tested, multiple testing corrections are not necessary. However, as technology improved and became more parallel, first with RNase protection assays and later with first-generation microarrays, this experimental design began showing weaknesses. Simulations using real and synthetic data confirmed that this sampling density is statistically underpowered (Atwood and Kay, 2012; Hughes et al., 2007, 2009) and contributed to marked lack of overlap in cycling genes detected by the first generation of circadian microarray experiments (Covington et al., 2008; Keegan et al., 2007; Wijnen et al., 2006).

For this reason, we recommend collecting samples at least every 2 h for studies of circadian rhythms, with more frequent sampling when studying ultradian rhythms (Hughes et al., 2009). This recommendation is based on down-sampling simulations of real data and on simulations using synthetic data (Atwood and Kay, 2012; Hughes et al., 2007, 2009, 2010). We acknowledge that this sampling scheme is not the current practice in the field, and we note that studies with relatively underpowered statistics can be valuable (1) when paired with extensive independent validation (Mizrak et al., 2012; Ruben et al., 2012), (2) when trailblazing a previously untested technology (Hughes et al., 2012), or (3) when screening a large number of samples with an expensive technology (Koike et al., 2012). As such, there is a trade-off between the time and money spent collecting additional samples up front and the amount of resources spent validating the hits from these experiments. In general, however, the evidence suggests that investigators should invest in more independent sampling to maximize the long-term utility of their data and the cost benefit of these experiments.

Although independent biological replicates increase statistical power, the high cost of “-omics” experiments can make it prohibitively expensive to collect replicate samples at each time point. Simulations indicate that replicates improve statistical power but are weaker than increasing temporal resolution if one is interested in estimating phase or amplitude (Hughes et al., 2010; Hutchison et al., 2015; see also the “Synthetic Data for Benchmarking” section). Therefore, good judgment must be used in choosing the right combination of replicates and temporal resolution for their intended application. ChIP-seq assays are an exception to this rule, since they tend to have greater variability between samples than other applications (Landt et al., 2012; Yang et al., 2014). As such, biological replicates at each time point are essential when performing ChIP-seq. Experiments on outbred organisms (such as humans) and samples collected in natural environments may also require independent biological replicates.

When using next-generation sequencing (RNA-seq, ChIP-seq, etc.), the depth of sequencing per sample should be explicitly considered in the planning stage. Greater sequencing depth costs more but results in better accuracy and precision. Finding the optimal cost/benefit ratio is not trivial, as the appropriate read-depth depends on the species studied, the size of the genome/transcriptome, the material from which libraries are prepared (e.g., polyA RNA or ribosome-depleted total RNA), the dynamic range of expression in a given tissue/species, and the strength of the circadian signal relative to noise. Oftentimes, it is advantageous to cull all features expressed below an empirically determined threshold to maximize statistical detection of bona fide cycling time series (Hughes et al., 2012; Menet et al., 2012; Soneson et al., 2016). For fly RNA-seq studies of total RNA, simulations show ~10 million reads are needed per sample to detect greater than 75% of truly rhythmic transcripts, while ~40 million reads per sample are needed for studying mammals (Li et al., 2015). These 2 reference points can be used to estimate read depths necessary in additional organisms based on the relative size of their transcriptomes. Although a comparable study has not been performed for ChIP-seq, the ENCODE consortium recommends 10 to 20 million mapped fragments per replicate in mammalian studies (Landt et al., 2012).

Variability in rhythmic profiles between individuals is an underexplored area in biological rhythms, particularly with respect to “-omics” technologies (Text Box 2.1). This is largely due to the nature of the experiments; for example, it is impossible to collect the suprachiasmatic nuclei (SCN) from an individual mouse more than once. Whenever feasible, serial collections from the same individual are ideal from a statistical perspective. When this is impossible, we recommend that studies of bulk circadian rhythms pool together as many different individuals as is practical (e.g., 5 or more individuals of the same gender) to average out variation between dissections and individuals. It is important to note that many studies have shown gender differences in circadian outputs such as locomotor activity rhythms, sleep, and even molecular rhythms. As such, some studies may benefit from analyzing the intraindividual variance in circadian rhythmicity. Given the ever-increasing multiplexing capabilities of new sequencing machines and the development of new technologies requiring less sequencing depth (Derr et al., 2016), it may soon become cost-effective and advantageous to analyze rhythmic gene expression in individuals (e.g., 3 to 5 individuals per time point).

**Figure:**
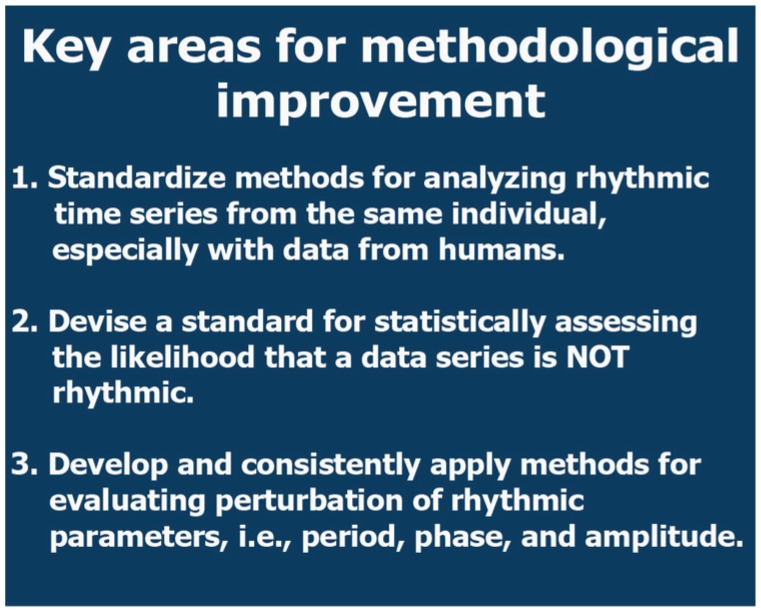


For human or other studies in outbred populations, we recommend sampling densities in excess of those typically used in laboratory model organisms to account for increased variability. Newly developed statistical methods, such as MetaCycle’s meta3d function and RAIN’s longitudinal mode, have been specifically developed to handle these time series data (Thaben and Westermark, 2014; Wu et al., 2016).

These recommendations apply to studies of nontraditional model organisms as well. Circadian rhythms are nearly ubiquitous among the kingdoms of life, and genome-scale techniques are being applied to circadian biology in new models. When practical, we recommend benchmarking new experimental systems using internal controls (i.e., genes, proteins, or processes known to be rhythmic in related species). For example, when measuring mRNA rhythms in a previously unstudied fungus, investigators would benefit from confirming that orthologs of known cycling genes such as *frq* and *wc-1* are rhythmic in their experiment. Bioinformatics approaches are under development to aid the discovery of clock gene orthologs in previously understudied species (Romanowski et al., 2014).

As discussed above, we emphasize that there is a trade-off between resources spent collecting the initial genome-scale data set and those spent in smaller-scale validation studies. For example, certain models are hard to breed (e.g., *Cry1/Cry2* double-null mice) or get enough of (e.g., VIP+ SCN neurons) or dangerous (e.g., serial sampling of solid tumors) to do a 2-h, 2-day time course. If the constraints of the experimental system necessitate a less rigorous experimental design, additional efforts should be made in follow-up experiments to validate the findings of the genome-scale analyses. At a minimum, follow-up experiments can be used to determine the empirical false discovery rate. Finally, when describing these experiments, the advantages and disadvantages of the experimental design and analysis methods should be acknowledged, and additional care should be given to their interpretation.

### Statistical Analysis

After generating a large data set, 3 steps should be taken to prepare the data for performing statistical analyses. The first is to verify the integrity of the raw files. For example, in RNA-seq experiments, this would include checking that the number of raw reads and quality scores are within appropriate ranges and that the expected numbers of unique and nonunique reads were detected (Hartley and Mullikin, 2015; Lohse et al., 2012). Checks for ribosomal, mitochondrial, chloroplast, or other contaminating sequences should be performed as well. Second, data should be normalized and quantified. This is the appropriate stage to check whether any internal controls agree with previous studies. For example, it is useful to check by eye whether known circadian clock genes are rhythmic with expected phase relationships. In human studies, it is valuable to confirm expected rhythms of melatonin and cortisol. For RNA-seq experiments, there are numerous methods for normalizing expression data, including situations under which the total amount of RNA per cell changes over time. These details are beyond the scope of this article, but we refer the interested reader to the relevant literature (Bray et al., 2016; Dobin et al., 2013; Schmidt and Schibler, 1995; Sinturel et al., 2017). In a ChIP-seq experiment, variation between samples is best handled by including multiple replicates and normalizing by randomly down-sampling the data (Koike et al., 2012). Third, data may need to be reformatted according to input requirements of the statistical methods used. For example, transcripts per million values in RNA-seq data should be log transformed before many statistical analyses.

There are numerous high-quality statistical approaches for detecting rhythmicity and estimating rhythmic parameters in large data sets. These include but are not limited to Haystack (Mockler et al., 2007), Lomb-Scargle (Glynn et al., 2006), ARSER (Yang and Su, 2010), CircWaveBatch (Oster et al., 2006), JTK_Cycle (Hughes et al., 2010), and its successors, RAIN (Thaben and Westermark, 2014), eJTK (Hutchison et al., 2015), and ABSR (Ren et al., 2016). Each has different strengths and weaknesses. To briefly summarize these methods, tests based on curve fitting such as COSOPT (Straume, 2004) are mathematically intuitive and work well but are underpowered and computationally inefficient (Hughes et al., 2010). Fourier analysis is popular but requires evenly sampled data and is limited in the period lengths it can detect (Wijnen et al., 2005). Analysis of variance can test for time-dependent changes, but it does not explicitly test for rhythmicity. JTK_Cycle is powerful and computationally efficient, but phase estimates are inaccurate when using sparse input data (e.g., less than every 4 h). Similarly, ARSER is powerful, but it does not consider replicates and cannot handle missing data. Certain algorithms (e.g., eJTK) perform better with replicates than repeated cycles (Hutchison et al., 2015). Many algorithms rely on an explicit or implicit fit to sinusoidal curves that may be problematic if the data include pulsed or asymmetric waveforms. We note that Haystack (Mockler et al., 2007) and ZeitZeiger (Hughey et al., 2016) are less sensitive to waveform shape than other algorithms. Some approaches are optimized for distinguishing ultradian rhythms from conventional 24-h rhythms (van der Veen and Gerkema, 2017). In many cases, however, investigators will have the greatest statistical power when searching for rhythms equal to conventional 24-h cycles. When studying clock mutants, free-running period should be measured with independent assays (e.g., free-running locomotor behavior) and statistical analyses of “-omics” data tuned to the appropriate organismal period length.

Since a full description of these attributes is beyond the scope of this article, we point the interested reader to previous studies that have tested these algorithms with benchmarking data sets (Deckard et al., 2013; Wu et al., 2014). Moreover, this is a rapidly changing field as newer approaches using machine learning (Agostinelli et al., 2016; Hughey, 2017; Laing et al., 2017) and N-version programming (Wu et al., 2016) have been recently developed that minimize some of the pitfalls described above. Time and implementation will tell which approaches are most valuable.

With algorithms, detecting more rhythmic features is not necessarily better, as both false-positive and false-negative observations are undesirable. The literature is rife with claims that each new algorithm detects more rhythmic components than previous methods. Although more sensitive detection is an understandable selling point, false-positives can be more costly than false-negatives. For example, a false-positive “hit” can result in a lab spending time and money following up on an ultimately unfruitful line of investigation. Therefore, we encourage the use of standardized, synthetic data for benchmarking the accuracy of each statistical method (see below) and rigorous empirical validation using independent experimental methods of any new discovery. When studying genome-scale rhythms, a conservative approach in declaring a given time series to be “rhythmic” is often appropriate.

Regardless of the statistical test being used, corrections for multiple testing are essential for genome-scale data (Qian and Huang, 2005; Text Box 1.2). The false-discovery rate (FDR) should be presented whenever discussing the number of rhythmic time series within any large data set (Hochberg and Benjamini, 1990; Macarthur, 2012; Storey et al., 2005). A typical microarray experiment measures upwards of 30,000 different transcripts; RNA-seq or ChIP-seq can measure millions of different abundances simultaneously. The dynamic range of mass spectrometer instruments limits the number of measurements made in proteomics and metabolomics, but tens of thousands of comparisons are common. The key insight when handling such large data is that even extremely unlikely patterns resulting in low *p* values become probable if enough measurements are taken. Therefore, one must always account for the size of the experiment and the number of statistical tests when presenting the confidence of a new discovery.

There is no correct statistical threshold at which to declare a time series “rhythmic” or “arrhythmic.” Therefore, we must reconcile ourselves to probabilistic answers. It is valuable to explore data using different statistical cutoffs, and we encourage investigators to show the number of cycling time series in a data set at different statistical thresholds. Alternatively, when considering individual time series, one can report how much variance is explained by a rhythmic function. When performing common downstream experiments based on lists of rhythmic components (e.g., pathway analysis), it is useful to verify that results are stable with respect to the statistical cutoff. Higher FDR thresholds may be advantageous in some cases, as overly restrictive cutoffs can disrupt the background gene set on which models of enrichment are based. Amplitude is another key consideration, as some rhythmic features may be of such low amplitude as to be biologically meaningless. We note that the field as a whole has frequently used “amplitude” and “fold change” interchangeably. Nevertheless, in many instances, the fold change—that is, the peak abundance divided by the trough abundance in a measurement—can be of essential biological significance. We therefore encourage investigators to explore filtering their data using amplitude, fold change, and/or the signal-to-noise ratio. Newer ontology analysis tools specific for biological rhythms such as phase set enrichment analysis (Zhang et al., 2016) may also be valuable in this context when exploring enriched pathways in rhythmic data sets.

The inherent imprecision of probabilistic results discussed above has important implications for the visual display of large-scale rhythmic data. For example, the ubiquitous Venn diagram comparing the number of rhythmic components in different data sets is often misleading since it simultaneously incorporates uncertainty from multiple independent experiments (Thaben and Westermark, 2016). As a result, Venn diagrams often overstate the differences between 2 or more experiments. Given how intuitively Venn diagrams display these results, it is unrealistic to expect them to disappear from the literature anytime soon. Nevertheless, we recommend enhancing the presentation of these data with several additional methods. For example, simple heat map representations of raw time-series data can be used to show whether the overall phase relationships and periodicity remain unchanged after a perturbation, although the underlying statistics may show different numbers of rhythmic components (for an example, see Xu et al., 2011). Even displayed en masse, there is great virtue in providing readers access to the raw, unmodified data. Similarly, directly comparing rhythmic parameters (Thaben and Westermark, 2016) of known cycling components (i.e., phase, period, amplitude) can yield more granular insight into the underlying result (for an example, see Atger et al., 2015). This is especially pertinent in cases in which the absolute level of expression of a feature may change dramatically in response to a perturbation. In short, we recommend against relying entirely on simple comparisons between the number of time series deemed to be rhythmic or arrhythmic by statistical analysis. Precisely how many rhythmic or arrhythmic features are found in a data set is a number that has no inherent biological importance.

Indeed, presuming that a given time series is arrhythmic based on a high *p* value is mathematically flawed. A high *p* value means that the observed data could have easily been generated under the null hypothesis, but it does not formally necessitate that the null hypothesis must be accepted. In addition, the confidence with which a data series is declared to be “rhythmic” depends on experimental details chosen by the investigator as discussed above. Simple binary divisions such as “rhythmic” and “arrhythmic” are thus capricious. In short, it is hard to define an “index of arrhythmicity” for time-series data using established tools. We note that the field could benefit from a more rigorous statistical definition of arrhythmicity (Text Box 2.2), perhaps based on how much of the variance in a time series is explained by rhythmicity.

An alternative to solving the significance problem is to focus on the assessment of rhythmic parameters such as period, phase, amplitude, and fold change. In many cases, the cardinal circadian parameters more accurately describe the underlying biological phenomena than abstract *p* values. However, we note that accurate and reliable estimation of rhythmic parameters is a different and tougher statistical challenge than simply determining whether a time series is rhythmic. Small changes in period length, for example, are often beyond the resolution offered by a typical “-omics” experiment. We encourage the development of more rigorous statistical methods for comparing rhythmic parameters and the more general use of existing tools. An expansion of JTK_Cycle took a first step toward this by calculating confidence intervals for amplitude measurements (Miyazaki et al., 2011). We note that this method relies on fitting the data to a cosine curve, which can be statistically problematic depending on the shape of the rhythmic time series (Janich et al., 2015). Furthermore, a recently released method called DODR (Thaben and Westermark, 2016) can be used for quantifying differences in rhythmic parameters. Taken together, we look forward to the field routinely using robust statistical methods for comparing perturbations of rhythmic parameters (Text Box 2.3).

### Synthetic Data for Benchmarking

**Figure 3.**
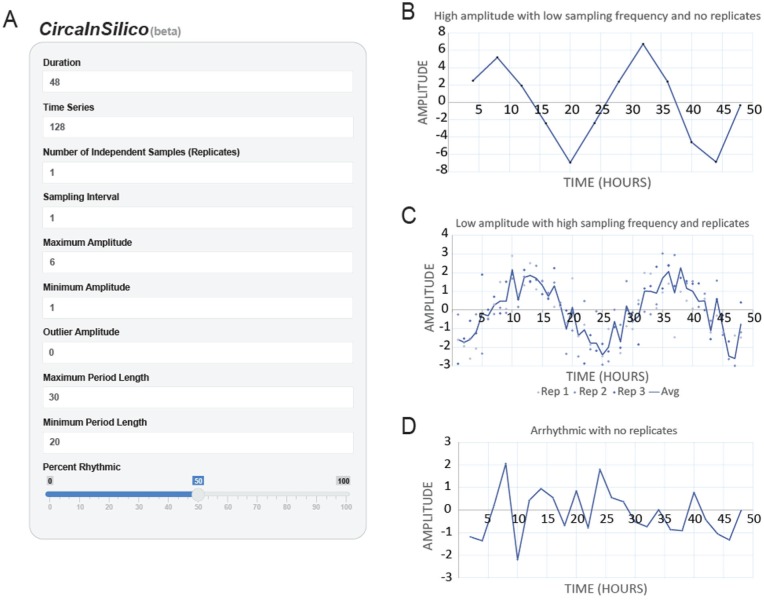
*CircaInSilico* generates synthetic time series for benchmarking analytical pipelines. (A) To simulate unique circadian data sets, *CircaInSilico* (https://5c077.shinyapps.io/Circa_in_Silico/) allows users to define the duration of the experiment, number of transcripts, number of replicates, amplitude range, period length, and the percentage of rhythmic transcripts. (B) High-amplitude rhythmic time series simulated by *CircaInSilico*. The duration of the experiment was set to 48 h with no replication and a sampling interval of 4 h. The period length of the transcript was 24 h, and the amplitude range was set to −7 and 7 (arbitrary units). (C) Low-amplitude rhythmic time series simulated by *CircaInSilico*. The duration of the experiment was set to 48 h, with a sampling interval of 1 h. The period length was set to 24 h, with an amplitude range from −3 to 3 (arbitrary units). Each time point was replicated 3 times, and the trend line represents the average expression at every time point. (D) Arrhythmic time series simulated by *CircaInSilico*. The duration of the experiment was set to 48 h with no replication and a sampling interval of 2 h.

As discussed above, there are many plausible experimental designs and statistical methods for identifying biological rhythms in large data sets. One way out of this wilderness is simply to test the empirical statistical power of different analytical pipelines. Here, we present CircaInSilico (https://5c077.shinyapps.io/Circa_in_Silico/), an online platform that allows users to generate data for simulating circadian experiments without requiring any a priori programming expertise (Fig. 3). Rhythmic and arrhythmic time series are sampled at user-defined intervals, and Gaussian noise is superimposed on the data to simulate technical and biological variance. Users can specify (1) the duration of the proposed data collection, (2) the total number of time series analyzed, (3) the number of replicates per time point, (4) the frequency of sample collection, (5) whether to include outlier data points, and (6) the percentage of time series that are genuinely rhythmic. The phases of rhythmic transcripts are uniformly distributed across the entire cycle, and period length and amplitude are uniformly distributed within user-defined ranges. These synthetic data are conveniently saved as *.csv files that include the true period length, phase, and amplitude.

Using this tool, investigators can systematically compare the statistical power of different analytical pipelines. To illustrate this, several example comparisons are supplied online, including how rhythmic identification depends on the duration and frequency of sample collection. The trade-off between sampling density and phase accuracy is also shown. We acknowledge that this tool is a starting point for further analyses, as it does not specifically simulate (1) batch effects, (2) uneven phase distributions, (3) trends and/or “red noise,” or (4) alternatively shaped rhythms, such as pulses or asymmetric waves. For this reason, a permanent copy of the source code for CircaInSilico is freely available on GitHub (https://github.com/5c077/Circa-in-Silico), and we encourage investigators to edit this code to fit their needs and to share with the field accordingly.

Simulations of statistical power are especially pertinent when proposing experiments to funding agencies that require justification for the number of vertebrate animals being used. If investigators can estimate parameters such as the animal-to-animal variance in measurements of gene, metabolite, or protein expression, they can simulate the expected data without spending any time or money on wet lab experiments. From these simulations, false-negative and false-discovery rates can be predicted for a range of different experimental designs, and an optimal number of vertebrate animals can be ascertained.

### Data Sharing

Published work must include all methodological details necessary for independent scientists to reproduce the results. This is particularly critical to genome-scale experiments, in which the enormity of the data ensures that even minor technical details can have a substantial impact on investigators reusing published results. Among these, quality or integrity metrics for input samples (e.g., RIN numbers for RNA) should be included in the methods. It is essential that any large-scale data in biological rhythms research be deposited in an appropriate, publically available database (Text Box 1.3). Data and analytical methods must be made available to peer reviewers to be downloaded anonymously; all data should be made public on acceptance of the manuscript. For the convenience of end users, .csv files with raw data and calculated *p* and *q* values are ideal. We support the International Society for Computational Biology’s stance that open data sharing is essential in modern biology (Berger et al., 2016), and we encourage the appropriate citation and acknowledgment of archived data sets. For functional genomic data sets (ChIP-seq, RNA-seq, ribosome profiling, methyl-seq, etc.), investigators typically deposit their data in NCBI’s Gene Expression Omnibus (GEO) or Sequence Read Archive (SRA). Proteomic data are typically deposited in the European Bioinformatics Institute (EMBL-EBI) proteomics database: PRoteomics IDEntifications (PRIDE). Metabolomic data are typically deposited in MetaboLights (EBI) or the Metabolomics Workbench (UCSD). Circadian-specific data sets can also be deposited in CircadiOmics (Patel et al., 2012). Similarly, it is recommended that authors upload all custom-built analytical methods to online repositories such as BitBucket, GitHub, or Sourceforge.

## Conclusions

When undertaking genome-scale analyses of biological rhythms, we must reconcile ourselves to probabilistic answers as opposed to simple binary (rhythmic or arrhythmic) classifications. Although systems biology has contributed enormously to our understanding of circadian rhythms, it also imposes huge costs in terms of time and money spent performing primary experiments and often much more in follow-up validation. Most critically, we need to ensure that these data contribute new insights into the underlying biological principles, rather than muddying the water with inaccurate or nonreproducible observations. A careful balance should be struck between the cost of an experimental design and the rigor and reproducibility of the results it can be expected to generate.

We recommend sampling at least 12 time points per cycle across 2 full cycles to optimize statistical power. Nevertheless, we acknowledge that many valuable studies have been performed with less rigorous designs. Certain particularly complicated or costly experiments may necessitate deviations from this guideline. These include but are not limited to (1) ecological studies of nonmodel organisms, (2) studies of human health and disease, (3) studies on aging, (4) pilot studies of new technical approaches, and (5) studies on especially costly or complicated breeds of mice. A key recommendation discussed above that applies to such studies is that there is a trade-off between discovery and validation, and explicit consideration of such issues in scientific reports will help to inform other researchers. In other words, additional efforts taken to validate novel findings can compensate for compromises made in the initial experimental design.

We propose 3 broadly applicable “golden rules” for conducting systems biology research on biological rhythms (Text Box 1). These guidelines will help ensure that published results properly account for the inherent uncertainty of such large-scale experiments and provide useful resources to future investigators. To date, the emphasis of these experiments has been in cataloging rhythmic profiles in different organisms and tissues. We believe that future progress in more accurately quantifying perturbations in systems-level rhythms (Text Box 2) will contribute to a deeper understanding of circadian output pathways and disease states. We emphasize that multiple technically independent lines of evidence are a universal solution to improve the reproducibility and reliability of any experimental discovery.
